# Synbiotic goat milk kefir improves health status in rats fed a high-fat and high-fructose diet

**DOI:** 10.14202/vetworld.2022.173-181

**Published:** 2022-01-28

**Authors:** Nurliyani Nurliyani, Eni Harmayani, Sunarti Sunarti

**Affiliations:** 1 Department of Animal Product Technology, Faculty of Animal Science, Universitas Gadjah Mada, Jl. Fauna 3, Kampus UGM, Bulaksumur, Yogyakarta 55281, Indonesia; 2 Department of Food and Agricultural Product Technology, Faculty of Agricultural Technology, Universitas Gadjah Mada, Jl. Flora 1 Bulaksumur, Yogyakarta 55281, Indonesia; 3 Department of Biochemistry, Faculty of Medicine, Public Health and Nursing, Universitas Gadjah Mada, Jl. Farmako, Senolowo, Sekip Utara, Yogyakarta 55281, Indonesia.

**Keywords:** health status, high fat-high-fructose diet, porang glucomannan, simvastatin, synbiotic kefir

## Abstract

**Background and Aim:**

Kefir, a natural probiotic containing bacteria and yeast, is a fermented milk product, whereas glucomannan from porang tuber (*Amorphophallus oncophyllus*) is prebiotic *in vivo*. Simvastatin is a potent lipid-lowering statin that can be utilized for pharmacological therapy in obesity. This study aimed to determine the effect of goat milk kefir supplemented with porang glucomannan (synbiotic kefir) and goat milk kefir without glucomannan (probiotic kefir) on blood glucose, hemoglobin A1c (HbA1c), free fatty acids (FFAs), tumor necrosis factor-alpha (TNF-α), gene expression of peroxisome proliferator-activated receptor gamma (PPARg), and insulin-producing cells in rats fed a high-fat and high-fructose (HFHF) diet.

**Materials and Methods:**

Male Sprague-Dawley rats were divided into five dietary groups: (1) Normal control, (2) rats fed HFHF, (3) rats fed HFHF+probiotic kefir, (4) rats fed HFHF+synbiotic kefir, and (5) rats fed HFHF+simvastatin. All of these treatments were administered for 4 weeks.

**Results:**

There were no significant differences in plasma glucose levels in HFHF diet-fed rats before and after treatment. However, plasma HbA1c and TNF-α decreased, and FFAs were inhibited in rats after treatment with synbiotic kefir. Synbiotic kefir decreased the gene expression of PPARγ2 in HFHF diet-fed rats but did not affect the total number of islets of Langerhans and insulin-producing cells.

**Conclusion:**

Synbiotic kefir improved the health of rats fed an HFHF diet by decreasing HbA1c, TNF-α, and PPARγ2 gene expression and preventing an increase in FFAs.

## Introduction

Limited physical activity and enhanced exposure to unhealthy foods and high energy may lead to obesity. Since 1975, obesity has nearly tripled worldwide. In 2016, more than 1.9 billion adults, 18 years and older, were overweight. Of these, over 650 million were obese. In 2020, 39 million children below 5 years old were overweight or obese [1]. In the past decade, the prevalence of obesity has become increasingly common and a major nutritional problem worldwide. Genetic factors and physiological problems also influence the risk factors in the development of obesity. Obesity may negatively affect the progression of insulin resistance, type 2 diabetes mellitus (T2DM), and metabolic syndrome. Because of the limited success of therapy to treat obesity and metabolic syndrome, an approach that requires interdisciplinary collaborations is needed to facilitate changes in eating behavior and physical activity [2]. Prevention and treatment of metabolic syndrome can be performed both pharmacologically and non-pharmacologically. Pharmacological therapy of obesity can be performed using lipid-lowering agents, such as statins, niacin, and bile acid sequestrants. Simvastatin is effective in lowering lipids levels through the suppression mechanisms of 3-hydroxy-3-methylglutaryl coenzyme A reductase, which plays a role in cholesterol biosynthesis [3]. Various approaches, including targeting lipoproteins, blood pressure, or anthropometric indices, can be employed to treat metabolic syndrome. The control of metabolism at the levels of lipoproteins and lipids is regulated by peroxisome proliferator-activated receptors (PPARs) [4]. Homeostasis of glucose, adipogenesis, and storage of lipids is controlled by PPAR gamma (PPARg), which is primarily expressed in the adipose tissue, and a minority is expressed in the macrophages and other cell types [5]. PPARg2 plays a significant role in adipogenesis, is a mediator in insulin sensitivity, is specific for adipose tissue [6], and is a potent transcription activator [7]. Functional food affecting health benefits can be derived from animal or plant sources. Kefir, a natural probiotic that contains lactic acid bacteria and yeast, is a fermented milk product [8,9]. Goat milk kefir is also more valuable than cow milk kefir because goat milk is easier to digest and has a higher mineral bioavailability and a more balanced protein and fat profile than cow’s milk [10,11].

A study by Choi *et al*. [12] demonstrated that kefir plays a significant role in reducing obesity in mice induced by a high-fat diet through weight loss and reduction of the epididymal fat layer and adiposity diameter, a decrease in the gene expression associated with adipogenesis and lipogenesis, and a decrease in the pro-inflammatory marker levels in epididymal fat. Recent study has demonstrated that kefir and isolated microorganisms have the potential to be antiatherosclerotic through an enhancement of anti-inflammatory cytokines and a reduction of pro-inflammatory responses [13]. Porang (*Amorphophallus oncophyllus*) is a local tuber that is often found in Indonesian forests and is being cultivated. Similar to *Amorphophallus konjac*, porang tuber contains glucomannan and has been demonstrated to be a prebiotic *in vivo* [14], which selectively enhances the growth of probiotic bacteria, such as lactobacilli and bifidobacteria [15]. Glucomannan is a water-soluble dietary fiber that can improve blood sugar, blood fat concentration, and weight management and provides other health benefits. Subjects with metabolic syndrome will be comfortable consuming glucomannan as a substitute for the main carbohydrates, and the risk of metabolic syndrome and oxidative stress can be reduced with the consumption of glucomannan noodles for 4 weeks [16]. The immunomodulatory effects of probiotics may originate from living and dead microorganisms [17], whereas the effect of prebiotic immunomodulation is associated with the growth stimulation of probiotics and their metabolites, such as short-chain fatty acids (SCFAs) and branched-chain fatty acids [18].

This study aimed to determine the effect of goat milk kefir supplemented with porang glucomannan (synbiotic kefir) and goat milk kefir without glucomannan (probiotic kefir) on blood glucose, hemoglobin A1c (HbA1c), free fatty acids (FFAs), tumor necrosis factor-alpha (TNF-α), gene expression of PPARg, and insulin-producing cells in rats fed a high-fat and high-fructose (HFHF) diet.

## Materials and Methods

### Ethical approval

The study was approved by the Medical and Health Research Ethics Committee, Faculty of Medicine Universitas Gadjah Mada, Indonesia (Approval number: KE/FK/95/EC/2015).

### Study period and location

The study was conducted from March to October 2016 at the Department of Animal Product Technolgy, Faculty of Animal Science, Universitas Gadjah Mada (UGM), the Integrated Research and Testing Laboratory, UGM, and the Department of Histology and Cell Biology, Faculty of Medicine, Public Health and Nursing, UGM.

### Kefir preparation

Synbiotic kefir was produced from a mixture of goat milk, porang glucomannan (as a prebiotic), whey protein concentrate (WPC), and kefir grain. Glucomannan from porang tuber was obtained from the Faculty of Agricultural Technology, Universitas Gadjah Mada, Yogyakarta, Indonesia. Fresh goat milk originated from Etawah crossbred goats in Yogyakarta, Indonesia. WPC was obtained from the Sari Husada Milk Industry in Yogyakarta, Indonesia. Kefir grain was purchased from a local supplier in Yogyakarta. Synbiotic kefir preparation was performed according to Otles and Çağındı [8] with slight modification. Goat milk, 0.1% WPC, and 0.3% porang glucomannan were mixed, pasteurized at 75°C for 15 min, and cooled at room temperature (27^o^C). Kefir grains (2%) were inoculated into pasteurized milk and incubated at 27^o^C for 18 h. After incubation, the kefir was filtered to separate kefir grains. Probiotic kefir was prepared using goat milk, WPC, and kefir grain without glucomannan. Synbiotic kefir was prepared by adding glucomannan to probiotic kefir.

### Animal experiments

Thirty male Sprague-Dawley rats (8-12 weeks old, body weight of around 200 g) were divided into five groups with six rats in each group: (1) normal control (negative control rats) that received a standard diet only, (2) rats fed an HFHF diet (positive control rats), (3) rats fed HFHF+probiotic kefir, (4) rats fed HFHF+synbiotic kefir, and (5) rats fed HFHF+simvastatin. The dose of kefir was 3.6 mL/200 g body weight/day for 4 weeks [19]. The range of recommended serving size per day of fermented milk for humans is 100-250 g according to Comerford *et al*. [20], whereas the dose of simvastatin for humans is 40 mg/day [21]. The dose conversion factor from humans to rats is 0.018 [22]. Therefore, the dose of kefir was 200 mL×0.018=3.6 mL, and the dose of simvastatin was 40 mg×0.018=0.72 mg. The rats were adapted to a standard AIN-93 diet (Table-1) [23,24] for 1 week and then treated with an HFHF diet (Table-1) for 2 weeks. The rats were administered with an HFHF diet until the end of the experiment (4 weeks). The composition of the standard and HFHF diets was prepared according to Reeves *et al*. [23] and de Castro *et al*. [24] with slight modification (Lard contained in the standard diet is replaced with beef tallow).

**Table-1:** Formulation of standard (AIN-93) and high-fat/high-fructose diets.

No.	Ingredient (g/kg)	AIN-93M*	High fat+fructose** (HFHF)
1	Fructose	-	321.6
2	Casein	140.00	190.25
3	Condensed milk	-	158
4	Soybean oil	40.00	20
5	Beef tallow (beef fat)	-	185
6	Fiber/Alphacel	50.00	25
7	Wheat bran	-	54.15
8	Mineral mix	35.00	35
9	Vitamin mix	10.00	10
10	DL-Methionine	1.80	1.8
11	Choline chloride	2.50	2.5
12	Corn starch	620.70	-
13	Sucrose	100.00	-

* Reeves *et al*. [23],

** de Castro [24]. HFHF=High fat and high fructose

### Blood analysis

Fasting plasma blood glucose was measured through an enzymatic photometric test using the glucose oxidase phenol 4-aminoantipyrine peroxidase method according to the instructions in the kit (DiaSys, Holzheim-Germany). Glycosylated HbA1c analysis was conducted using Rat HbA1c ELISA Kit (Elabscience, Wuhan, China), according to the manufacturer’s protocols. Analysis of plasma FFAs was conducted using Rat FFA ELISA Kit (Qayee-Bio, Shanghai, China) following the manufacturer’s instructions. Plasma TNF-α was analyzed using rat-specific ELISA kits for the measurement of TNF-α following the manufacturer’s instructions (eBioscience, Bender MedSystems, Vienna, Austria).

### Gene expression analysis

PPARγ2 gene expression was analyzed through the following four steps: (1) RNA isolation from white adipose and liver tissues, (2) reverse transcription from RNA to cDNA using reverse transcriptase enzyme, (3) cDNA amplification by polymerase chain reaction (PCR), and (4) quantification and detection of cDNA products using real-time PCR.

Total RNA was extracted from the adipose and liver tissues using TRIzol reagent (Sigma-Aldrich, USA), and the mRNA levels were analyzed using real-time PCR. Reverse transcription of total RNA was performed using the Transcriptor First Strand cDNA Synthesis Kit (Roche Diagnostics, Germany) to produce cDNA. Real-time PCR was performed in a mixture (final volume of 20 μL) containing 2 μL of cDNA (DNA template), 10 μL of EvaGreen (Biotium, Inc., USA), 1 μL of glyceraldehyde-3-phosphate dehydrogenase (GAPDH), and 6 μL of RNAse-free water. Likewise, PPARγ2 was reverse transcribed with additional reagents totaling 20 μL. The mRNA amount was calculated as the ratio to the value of GAPDH in each cDNA sample. The primary nucleotide sequences used to detect each mRNA were designed using Primer Express Software v3.0.1 (Thermo Fisher, USA) according to the sequences available in the GenBank database [25]. The primary nucleotide sequences are presented in Table-2.

**Table-2:** Primer nucleotide sequences for real-time PCR.

Gene	Primer		Length of PCR product (bp)	GenBank accession no.
PPARγ2	Sense	5′-ACTCTGGGAGATCCTCCTGTTG-3′	68	Y12882
	Antisense	5′-GAAGTGCTCATAGGCAGTGCAT-3		
GAPDH	Sense	GCC GAG GGC CCA CTA AAG	70	BC059110
	Antisense	TGC TGT TGA AGT CAC AGG AGA CA		

Optimization of cDNA amplification products was performed using conventional PCR with a program at a temperature of 95°C for 5 min, 95°C for 1 min, 58°C for 1 min, and 72°C for 1 min with 34 cycles. The temperature was maintained at 72°C for 5 min and 12°C for 5 min. The optimized program for real-time PCR was established to be 95°C for 5 min, 95°C for 1 min, 60°C for 30 s, and 72°C for 1 min for 39 cycles. The melt curve was maintained at 65°C-95°C for 5 s, and then, the plate was read. The average change in the level of gene expression (2^−ΔΔCT^) of PPARγ2 was analyzed according to Livak and Schmittgen [26].

### Immunohistochemistry of insulin-producing cells

Pancreatic tissue was prepared for the immunohistochemical (IHC) staining of insulin-producing β-cells. The slides were deparaffinized with xylene and hydration with ethanol and immersed in a glass jar containing 0.3% H_2_O_2_ in methanol. H_2_O_2_ was removed, and the slides were washed with distilled water 3 times and then washed with phosphate-buffered saline (PBS) 3 times. The slides were placed in a humid chamber, incubated for 10 min, and then incubated with mouse monoclonal insulin primary antibody (dilution 1:1000) (Abcam, [K36aC10] ab6995, Cambridge, USA) for 30 min. Then, the slides were washed with PBS 3 times, incubated with secondary antibody in a humid chamber for 10 min, and then washed with PBS 3 times. The slides were placed in a humid chamber to be incubated with streptavidin horseradish peroxidase for 10 min. Subsequently, the slides were washed with PBS 3 times and incubated with 3,3’-diaminobenzidine substrate (dilution 1:50) for 15 min in a dark humid chamber. The slides were washed with distilled water 5 times, counterstained with hematoxylin, mounted with coverslips, and observed under a light microscope. The number of islets of Langerhans and insulin-positive β-cells was counted using a colony counter and documented using an Opti Lab (SOP No. A-007) microscope. The number of islets of Langerhans and insulin-producing β-cells in each rat was averaged from three fields of view.

### Statistical analysis

Data from this analysis are expressed as mean±standard deviation. Before and after treatments, blood plasma analysis data included fasting blood glucose, HbA1c, FFAs, and TNF-α. The difference between the mean of blood plasma analysis before and after treatments was analyzed using the paired samples t-test. Statistical analyses of the PPARγ2 gene expression, the total number of islets of Langerhans, and the total number of insulin-producing cells were conducted using one-way analysis of variance followed by Duncan’s multiple range test (p<0.05 indicated significant differences). Statistical analyses were performed using SPSS version 17.0 software (IBM Corp., NY, USA).

## Results

### Blood glucose

Table-3 demonstrates that the blood glucose levels were still within the normal glucose range, and there was no difference before and after treatment in the negative controls. Rats that received only the HFHF diet (positive controls) exhibited higher glucose levels after treatment (after being given the diet for 5 weeks) than those before treatment, but the increase was not significant. Goat milk kefir supplemented with porang glucomannan could reduce blood glucose levels, but the decrease, which was only 11.9 mg/dL, was not significant. Treatment with simvastatin significantly reduced blood glucose levels in rats fed an HFHF diet by approximately 139.02 mg/dL.

**Table-3:** The average blood glucose in rats before and after treatments.

Treatments	Fasting blood glucose (mg/dL)	

	Before treatment	After treatment
Normal control	94.11±19.02^a^	95.51±21.10^a^
HFHF	104.05±12.93^a^	118.90±11.33^a^
HFHF+probiotic kefir	112.81±9.54^a^	114.34±18.81^a^
HFHF+synbiotic kefir	116.98±6.76^a^	105.08±11.93^a^
HFHF+simvastatin	221.37±6.76^a^	82.35±11.93^b^

Different letters in the same row indicate significant differences (p<0.05). HFHF=High fat and high fructose

### HbA1c

Table-4 demonstrates that the HbA1c levels in rats after treatment with synbiotic kefir were lower than those before treatment (p<0.05). However, other groups, including those who received probiotic kefir treatment, did not significantly differ before and after treatment.

**Table-4:** The average HbA1c in rats before and after treatment.

Treatments	HbA1c (ng/mL)	

	Before treatment	After treatment
Normal control	21.47±5.18^a^	24.33±3.35^a^
HFHF	24.98±2.92^a^	26.45±4.60^a^
HFHF+probiotic kefir	26.02±4.79^a^	35.44±18.99^a^
HFHF+synbiotic kefir	28.89±4.12^a^	23.56±3.47^b^
HFHF+simvastatin	22.72±4.64^a^	33.61±16.45^a^

Different letters in the same row indicate significant differences (p<0.05). HFHF=High fat and high fructose

### FFAs

Table-5 demonstrates that the average plasma FFA levels in rats after various treatments were higher in all groups than before treatment (p<0.05), but the increase in FFAs after kefir treatment was not significant.

**Table-5:** The average plasma FFAs in rats before and after various treatments.

Treatments	FFAs (ng/mL)	

	Before treatment	After treatment
Normal control	54.20±5.47^a^	61.41±2.19^b^
HFHF	60.15±4.66^a^	63.25±3.74^b^
HFHF+probiotic kefir	59.92±2.74^a^	62.24±2.84^a^
HFHF+synbiotic kefir	59.59±4.49^a^	62.85±4.13^a^
HFHF+simvastatin	54.04±8.32^a^	61.21±6.21^b^

Different letters in the same row indicate significant differences (p<0.05). HFHF=High fat and high fructose

### TNF-α

Table-6 demonstrates that, after various treatments, there was no decrease in the TNF-α levels in rats, except in rats treated with synbiotic kefir.

**Table-6:** The average plasma TNF-α in rats before and after treatment.

Treatments	TNF-α (pg/mL)	

	Before treatment	After treatment
Normal control	157.66±15.71^a^	166.66±20.84^a^
HFHF	170.33±23.54^a^	271.33±167.86^a^
HFHF+probiotic kefir	159.33±14.06^a^	208.00±44.68^b^
HFHF+synbiotic kefir	176.50±13.79^a^	155.00±6.63^b^
HFHF+simvastatin	169.33±11.07^a^	192.33±50.49^a^

Different letters in the same row indicate significant differences (p<0.05). HFHF=High fat and high fructose, TNF-α=Tumor necrosis factor-alpha

### PPARγ2 gene expression

The average change in the level of PPARγ2 gene expression (2^−ΔΔCT^) in white adipose tissue from the HFHF diet-fed rats treated with kefir with or without glucomannan was not significantly different from that of rats treated with simvastatin. The rats treated with kefir had a lower change in PPARγ2 gene expression than the HFHF diet-fed rats without kefir (p<0.05) (Table-7).

**Table-7:** Average relative expression of the PPARγ2 gene in white adipose (WAP) and liver tissue from rats receiving various treatments.

Treatments	PPARγ2 gene in WAP			PPARγ2 gene in liver		
	
	D CT	DD CT	2^−ΔΔCT^	D CT	DD CT	2^−ΔΔCT^
HFHF	−3.74±1.08^a^	−0.67±1.08^a^	1.96±1.24^a^	0.00±1.59^a^	−6.28±1.59^a^	123.46±120.56^a^
HFHF+probiotic kefir	−2.41±1.12^ab^	0.65±1.12^ab^	0.80±0.55^b^	1.88±1.12^b^	−4.41±1.12^b^	27.25±21.01^b^
HFHF+synbiotic kefir	−1.00±0.94^bc^	2.06±0.94^bc^	0.29±0.23^b^	3.84±0.89^c^	−2.44±0.89^c^	6.33±3.78^b^
HFHF+simvastatin	−2.22±1.12^c^	0.66±1.12^c^	0.70±0.52^b^	2.77±1.97^bc^	−3.51±1.99^bc^	19.79±17.89^b^

Different letters in the same column indicate significant differences (p<0.05). In WAP tissue: Normal control rats had an average DCT of –3.07, an average DDCT of 0.00, and an average change in the gene expression of PPARγ2 (2^−ΔΔCT^) of 1.00. In liver tissue: Normal control rats had an average DCT of 6.29, DDCT of 0.00, and an average change in the gene expression of PPARγ2 (2^−ΔΔCT^) of 1.00. PPARγ2=Peroxisome proliferator-activated receptor gamma 2, HFHF: High fat high fructose

Table-7 demonstrates that the change in the PPARγ2 gene expression in the liver tissue was greater than that in the adipose tissue. In the rats fed an HFHF diet without kefir supplementation, the greatest changes in the PPARγ2 gene expression (p<0.05) were observed in adipose and liver tissues. The simvastatin-treated rats had lower changes in PPARγ2 gene expression (p<0.05) than HFHF diet-fed rats.

### IHC staining of β-cells

**Figure-1:**
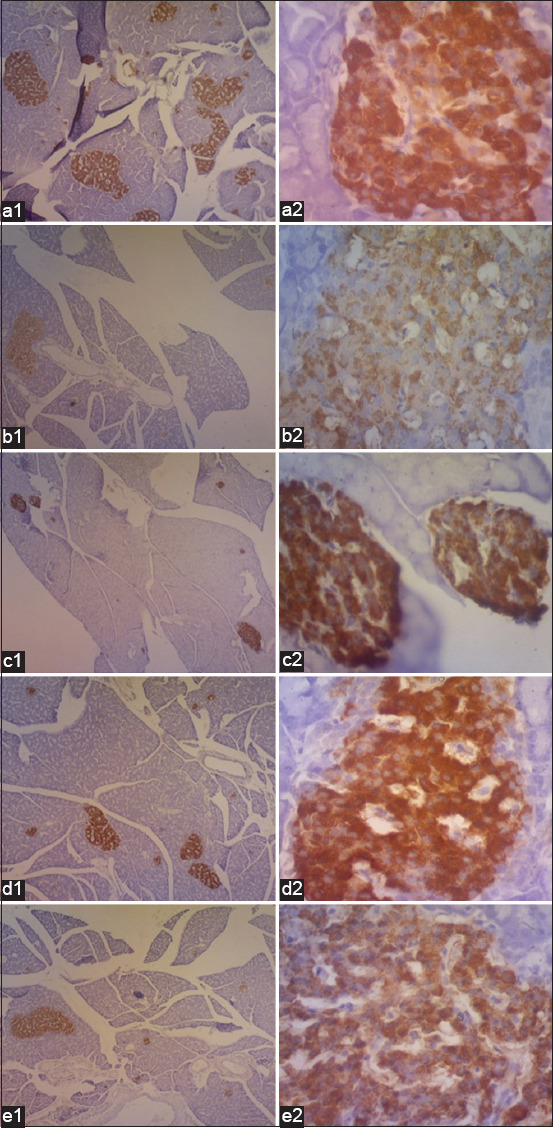
Immunohistochemistry of insulin-producing β-cells (brown color). a: Normal control; b: High fat high fructose (HFHF); c: HFHF+probiotic kefir; d: HFHF+synbiotic kefir; e: HFHF+simvastatin. a1-e1: 40× and a2-e2: 400×.

Figure-1 shows that, in HFHF-fed rats, Langerhans islet staining was rarely observed, and very weak IHC staining intensity for insulin-producing β-cells and few insulin-producing β-cells was observed. The HFHF diet-fed rats given simvastatin exhibited less strong IHC staining intensity than those with kefir treatment. However, rats fed an HFHF diet with probiotic or synbiotic kefir showed a strong color intensity of IHC staining, as in normal rats.

Table-8 shows the average numbers of Langerhans islets and insulin-producing β-cells with various treatments. The average numbers of Langerhans islets and insulin-producing β-cells in rats administered an HFHF diet without kefir supplementation demonstrated the lowest numbers, although these were not significantly different.

**Table-8:** The average numbers of Langerhans islets and insulin-positive β-cells in rats with various treatments.

Treatments	Langerhans^ns^	Insulin-positive β-cells^ns^
Normal control	3.04±0.78	96.95±94.19
HFHF	2.47±0.76	46.08±2.59
HFHF+probiotic kefir	2.90±0.71	71.74±22.42
HFHF+synbiotic kefir	3.55±0.65	82.14±45.27
HFHF+simvastatin	3.05±0.82	107.35±79.95

ns=Non-significant, HFHF: High fat high fructose

## Discussion

All rats fed an HFHF diet demonstrated a risk factor for metabolic syndrome with fasting blood glucose >100 mg/dL [4]. In this study, no decrease in blood glucose was observed after kefir treatments (probiotic and synbiotic kefir) in rats fed the HFHF diet, except for those treated with simvastatin. However, a konjac-derived glucomannan supplement (3.6 g/day) administered for 28 days reduced the blood lipid and glucose levels by increasing fecal excretion of neutral sterol and bile salt and decreased the enhanced glucose concentrations in subjects with hyperglycemic diabetes [27]. Contrary to the finding of a previous study [19], skim milk kefir given at a dose of 3.6 mL/day for 4 weeks could significantly reduce blood glucose levels by 111.00 mg/dL. In the present study, the small decrease in blood glucose was possible because the synbiotic kefir dose was still insufficient to play a role in reducing blood glucose in rats that consumed an HFHF diet during the experiment. In a previous study [19], diabetic rats were not fed an HFHF diet. The low dose of glucomannan in kefir and the difference in the conditions of the subjects may not cause a significant reduction in the blood glucose levels.

The decrease in blood glucose by simvastatin treatment in this study is in line with a previous study [28], in which mice fed a high-fat diet and administered with rosuvastatin exhibited lower blood glucose, which might be due to improved glucose uptake; however, β-cell activity is inhibited through lowered insulin levels and inhibited Ca^2+^ signaling in β-cells, resulting in reduced insulin secretion. The effects of rosuvastatin on glucose homeostasis are 2-fold: Increased insulin sensitivity, whereas β-cell activity is inhibited. In another study [29], glucose uptake in the adipose tissue was upregulated in pravastatin-treated mice fed a high-fat/high-sucrose diet and db/db mice. Contrary to the findings of previous studies [30,31], simvastatin can increase the risk of T2DM, particularly in subjects with pre-diabetes, due to hyperglycemia by decreasing the function of islet β-cells and exerts a negative effect on glucose homeostasis, particularly on fasting blood glucose levels. A high dose of atorvastatin impairs glycemic regulation in patients with DM [32]. According to Kim *et al*. [33], glucose metabolism may be affected by different kinds of statins. These studies indicated that the possible effect of statins on blood glucose levels depends on the dose and type of statin and the condition of the subject used for the study.

Porang glucomannan added to kefir could improve glucose metabolism to reduce glycosylated Hb. A previous study [34] demonstrated that the synergistic effects of these two components, namely, probiotics and prebiotics, make it a more effective supplement than probiotics or prebiotics separately. According to Patel *et al*. [35], the high-fructose feeding related to the present study could be a major risk factor for diabetes complications, including the development of hyperglycemia, insulin resistance, hyperinsulinemia, and hypertriglyceridemia, due to changes in carbohydrate and lipid metabolism as well as liver inflammation caused by fructose metabolism in the liver. In addition, type 2 diabetes complications, such as high blood glucose, glycosylated HbA1c, cholesterol, triglycerides, and oxidative stress, can be induced by the administration of high fructose for a long time (63 days) [36]. However, supplementation with fermented milk containing the probiotic *Lactobacillus rhamnosus* GG (150 g/kg standard diet) can mitigate the increase in glycosylated HbA1c in rats induced by diabetes by feeding a high-fructose diet [37]. A previous study [16] reported that 24 individuals with T2DM had significantly decreased HbA1c by 7.7% after consumption of glucomannan noodles.

In this study, the probiotic and synbiotic kefir maintained the plasma FFA levels in HFHF rats. The previous studies reported that konjac-glucomannan supplementation (5%) in baboons resulted in a relatively lower reduction in the initial values of triglycerides and circulating FFAs after 9 weeks [37,38]. The lower dose of glucomannan from porang tuber in the present study compared with the previous study [38] resulted in no decrease in plasma FFAs. According to Venter *et al*. [38], increased levels of circulating FFAs can stimulate fibrinogen synthesis in the liver. Elevated plasma fibrinogen is characteristic of insulin resistance in the liver (the synthesis of fibrinogen may be controlled by insulin). Furthermore, glucomannan from konjac, which is fermented in the colon, can decrease FFA production, including propionate production, which leads to a decrease in fibrinogen synthesis. Therefore, the metabolic effect of fiber is influenced by the outcome of the colony and SCFA absorption originating from soluble fiber [37]. Each kind of FFA can affect various physiological processes, such as the control of lipolysis and lipogenesis in the adipose tissue, inflammation, endocrine signaling, and compounds and characteristics of cellular membranes. The progression of insulin resistance and coagulatory damage may result from the physiological changes caused by changed plasma FFA levels or profiles [39].

In this study, porang glucomannan added to kefir may reduce inflammation by decreasing the pro-inflammatory cytokine production in rats administered an HFHF diet. The effect of soluble fiber in porang glucomannan on the improvement of metabolic disorders is in accordance with a previous study using chitosan fiber [40], which was given to rats with metabolic disorders (induced by diabetes); such a study demonstrated that the treatment can improve insulin resistance and chronic inflammation through decreased lipid absorption and slow down the absorption of glucose in the small intestine after eating, resulting in a decrease in hepatic lipids and adipose tissue weight and reduction of plasma adipocytokine levels, including leptin, TNF-α, and plasminogen activator inhibitor-1. In addition, according to Zhai *et al*. [41], konjac-glucomannan combined with bacterial cellulose exerted a better effect on liver inflammation due to obesity by lowering the levels of TNF and IL-6 and reducing the protein expression of nuclear factor erythroid 2-related factor 2 compared with mice that were only given additional bacterial cellulose or konjac-glucomannan alone. Furthermore, the combination of glucomannan and spirulina blocks the detrimental effects promoted by a hypercholesterolemic diet in Zucker rats, which could decrease the plasma TNF-α levels as an inflammation biomarker [42].

Peroxisome proliferator-activated receptor gamma-2 is most abundantly expressed in adipocytes and plays major adipogenic and lipogenic roles in the tissue (a main role in the differentiation and proliferation of adipose tissues) [43]. In the present study, the rats received an HFHF diet, it might have caused fatty liver. According to Lakhani *et al*. [44], mice fed a high-fat diet exhibited high PPARg expression in the liver. The change in the gene expression in the present study was lowest in the tissue of rat treated with synbiotic kefir. However, this difference was not significant compared with probiotic kefir treatment. Kefir-containing probiotics may synergize with the prebiotic glucomannan and play a role in the downregulation of PPARγ2 expression in white adipose and hepatic tissues. The study results were in line with those of a previous study [12], which reported that mice fed a high-fat diet supplemented with 0.2% kefir powder for 8 weeks had lower PPARg gene expression in the epididymal fat. In another study, mice fed a high-fat diet and 1 × 10^7^ or 1 × 10^9^ CFU/mouse probiotic *Lactobacillus plantarum* LG42 supplementation daily for 12 weeks had reduced PPARg expression in the adipose tissue [45], whereas *L. plantarum* K21 intervention (10^9^ CFU/day for 8 weeks) reduced the expression of PPARg in the liver tissue. After high-fructose treatment, reduced levels of PPARg and GLUT4 mRNA were also enhanced by the administration of *Lactobacillus reuteri* GMNL-263 [46].

Dietary fiber intake, mainly combined with bacterial cellulose/konjac-glucomannan, increases the antioxidant defense system and lowers lipid peroxidation in the liver by enhancing the activity of antioxidant enzymes and lowering the production of malondialdehyde in the liver. Furthermore, combined supplementation with bacterial cellulose and konjac glucomannan regulated the levels of leptin and adiponectin. It inhibited the protein expression of PPARg by reducing the size of cells in the adipose tissue of high fat diet-fed mice [41]. According to Han *et al*. [47], the lowering the weight of adipose tissue in rats fed biocellulose may be due to the reduction of adipocytes size, suggesting that biocellulose could suppress the hypertrophy of adipocytes, and thus may be very useful for the control of obesity. In the development of obesity, the size of adipocytes is enlarged due to the accumulation of fat. As body weight decreases, the size of adipocytes also decreases due to the reduction of fat.

Leptin and adiponectin are adipokines expressed and secreted by adipose tissue [48]. Leptin, regulates adipose mass and body weight by inhibiting food intake and stimulating energy expenditure [49], whereas adiponectin plays a vital role in improving obesity and metabolic diseases, and it is induced during adipocyte differentiation. Meanwhile, PPARg is the main regulator of adiponectin expression and processing [50]. According to Mi *et al*. [49], leptin was enhanced and adiponectin lowered in overweight/obesity, with adiponectin reducing with puberty (boys and girls) and leptin enhancing with puberty (girls).

The greatest changes in PPARγ2 gene expression in both the adipose and liver tissues of rats treated with HFHF without kefir in the present study were in accordance with the results in a previous study [51] that found that the PPARg expression level was significantly higher in rats fed a high-fat diet than in those fed a normal diet, which is mainly related to fat formation. It has also been explained that the expression of PPARγ2 in the liver, mainly in the hepatocytes, is positively correlated with fat accumulation due to diseases such as obesity and diabetes [43].

In this study, no change in the number of islets of Langerhans and insulin-producing β-cells was observed in all treatments, indicating that an HFHF diet received during the experiment did not cause β-cell damage. This was also evidenced by the unchanged average fasting blood glucose levels in the HFHF diet-fed rats before and after being treated with kefir (Table-2). According to Linnemann *et al*. [52], a decrease in pancreatic β-cell mass occurs in individuals suffering from T2DM, and fasting blood glucose will increase if the volume (mass) of cells is less than the 1.1% threshold [53]. If it is below this threshold value, changes in insulin sensitivity and functional damage in insulin production will significantly impact blood glucose. In our study, the HFHF diet had not yet led to diabetes but only caused pre-diabetes as the concentration of blood glucose ranged from 100 to 125 mg/dL, which is at risk of progressing to diabetes (>126 mg/dL), whereas normal blood glucose was <100 mg/dL [54,55]. The total percentage of fat content in the HFHF diet in the present study was approximately 21.76% (2% fat from soy oil, 1.26% fat from condensed milk, and 18.5% fat from tallow). According to Gulen *et al*. [56], damage to the islets of Langerhans occurred in rats fed 45% and 60% fat diets acutely (for 3 weeks) or chronically (for 8 weeks), as well as with chronic administration of the 45% fat diet. Therefore, no damage to the islets of Langerhans was observed in the present study when the dietary fat level was only 21.76% for less than 8 weeks.

IHC staining of pancreatic tissue revealed that insulin-producing β-cells stained brown when rat anti-insulin antibodies were used (Figure-1). The lower intensity in the IHC staining of HFHF-fed rats in line with the findings of the previous study in which rats were treated with a 45% fat diet chronically showed a decrease in the intensity of IHC staining [56]. In Figure-1, a strong color intensity of IHC staining in rats fed HFHF with probiotic or synbiotic kefir indicates that probiotic microorganisms in kefir play a significant role in improving insulin-producing β-cells. This finding was supported by the previous study on diabetic rats treated with konjac extract (containing glucomannan) alone, which showed less improvement of insulin-producing β-cells [57].

## Conclusion

The poor health status caused by the habit of consuming HFHF diets can be improved by consuming synbiotic goat milk kefir, which decreases the HbA1c, TNF-α, and PPARγ2 gene expression and prevents the increase in FFAs. This study demonstrates the possibility of preventing and treating metabolic disorders using natural substances, such as animal bioresources. Therefore, the application of synbiotic goat milk kefir containing porang glucomannan is suggested in the food industry to develop synbiotic-based functional foods that have the potential to improve health.

## Authors’ Contributions

NN: Planned and performed the study and drafted the manuscript. EH: Planned the study and analyzed the samples in the laboratory. SS: Conducted animal experiment and sampling. All authors have read and approved the final manuscript.
